# Environmental impact of portable MRI in remote Canadian settings

**DOI:** 10.3389/fnimg.2026.1706184

**Published:** 2026-08-28

**Authors:** Nawab Azizi, Zier Zhou, Alexander Ibrahim, Omar Islam

**Affiliations:** 1 School of Medicine, Queen’s University, Kingston, ON, Canada; 2 Department of Diagnostic Radiology, Kingston Health Sciences Centre, Queen’s University, Kingston, ON, Canada

**Keywords:** carbon emissions, energy, portable MRI, transportation, waste, remote, rural, community

## Abstract

**Background:**

Patients in remote communities often lack access to magnetic resonance imaging (MRI), necessitating long-distance transfers to tertiary centres for neuroimaging. Portable MRI (pMRI) addresses this gap by enabling bedside imaging without conventional scanner infrastructure. Its environmental profile, including energy consumption, patient transportation, and waste generation, compared with fixed MRI systems has not been comprehensively quantified in the literature.

**Methods:**

We conducted a mixed-methods analysis of an ultra-low-field pMRI scanner, focusing on three domains: (i) energy consumption, (ii) transportation-related greenhouse gas (GHG) emissions, and (iii) waste generation. Published data on energy use and emissions from 1.5 Tesla (T) and 3 T scanners were compared with modelled estimates for pMRI. Transportation emissions were calculated for patient travel between Weeneebayko General Hospital (Moose Factory, Ontario) and Kingston Health Sciences Centre (Kingston, Ontario) using commercial, charter, and air ambulance flights. Waste-related emissions were assessed based on shielding materials, helium consumption, and gadolinium-based contrast agent use.

**Results:**

pMRI consumed 11.8–17.4-fold less energy per scan than fixed 1.5 T and 3 T systems, corresponding to approximately 0.065 tons (t) CO₂ annually in a modelled 100–scan/year scenario. Based on published estimates, 56% of patients could avoid inter-facility transfer, which is estimated to reduce 14.3 t CO₂ emissions per year, equivalent to emissions from ~1.9 single-family homes. Waste analyses demonstrated that pMRI eliminated helium use, reduced shielding requirements, and minimized contrast-related waste, together preventing an additional ~5.4–9.8 t CO₂ per scanner over its lifecycle, which was equivalent to emissions produced by ~0.73–1.3 homes annually. This conservatively equates to a total carbon emissions reduction of 19.7–24.3 t per year or 2.6–3.1 home equivalents per year with pMRI implementation.

**Conclusion:**

In a remote Canadian setting, pMRI provided local neuroimaging access to a predominantly Indigenous population, with 56% of patients avoiding inter-facility transfer while maintaining diagnostic quality. These clinical benefits were accompanied by a markedly reduced carbon footprint, supporting pMRI as both a health-equity intervention and a sustainable imaging strategy for underserved, remote regions.

## Introduction

The healthcare sector has come under scrutiny for its environmental impacts, as medical services contribute substantially to global greenhouse gas (GHG) emissions and waste generation (Anudjo et al., 2023). Globally, healthcare systems account for an estimated 5.0–8.5% of GHG emissions, underscoring the urgency of adopting more sustainable practices (Hanneman et al., 2024a). Within radiology, magnetic resonance imaging (MRI) is indispensable for the diagnosis and management of a wide range of conditions due to its ability to provide high-resolution, soft-tissue visualization without ionizing radiation. However, fixed MRI systems are resource-intensive, with considerable energy demands and associated waste streams, including helium consumption, electronic waste, and environmental contamination from gadolinium-based contrast agents (Heye et al., 2020; Chaban et al., 2023). These factors have prompted calls to optimize imaging practices through scanner efficiency, reduced repeat imaging, and greater integration of digital technologies such as telemedicine (Hanneman et al., 2024b; Health Canada, 2023).

**Figure 1:**
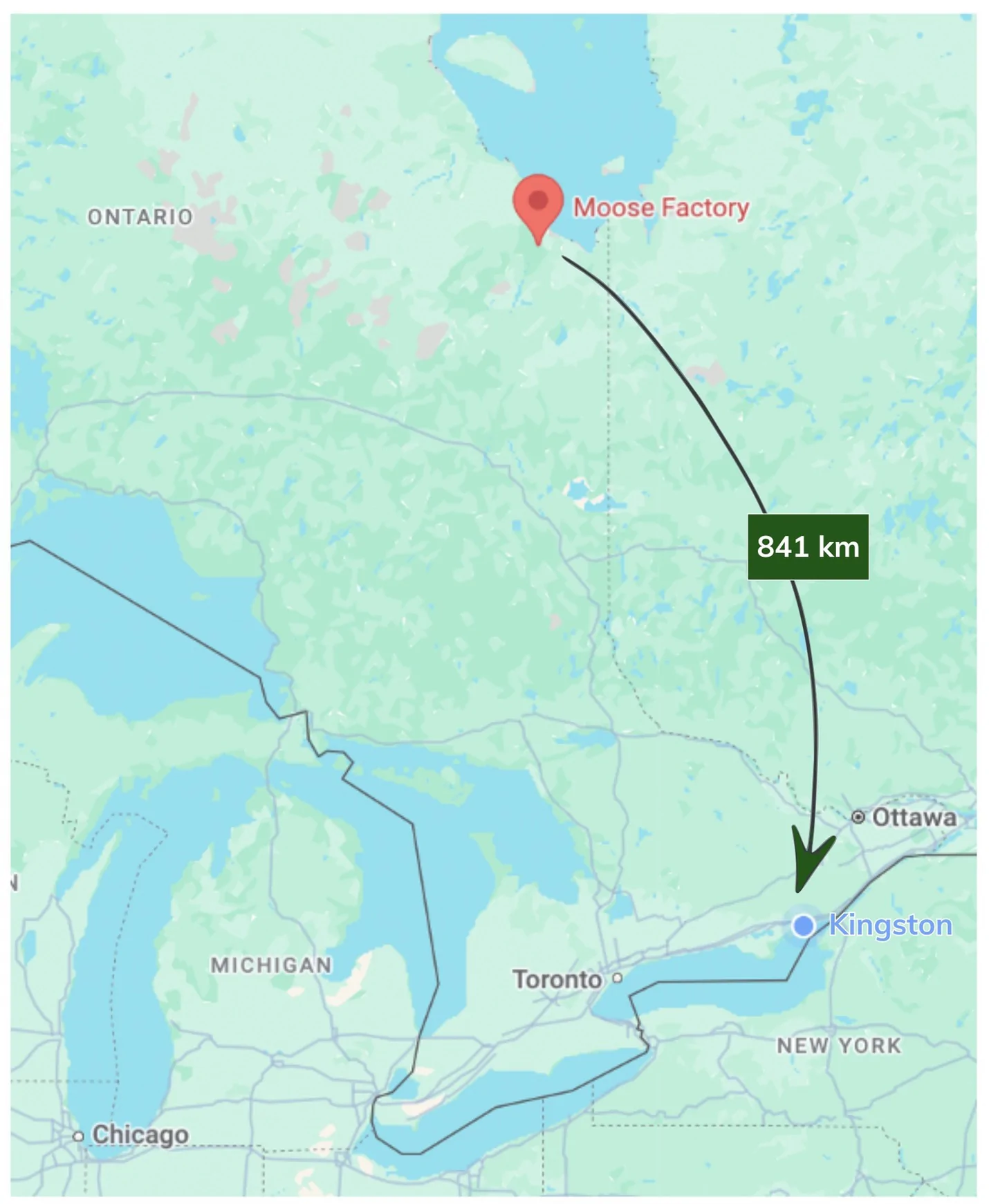
Map illustrating the distance (841 km) that Weeneebayko General Hospital patients from Moose Factory, Ontario must travel to receive MRI imaging at Kingston General Hospital in Kingston, Ontario.

Beyond the direct clinical burden of imaging equipment, patient transportation to centralized radiology services imposes considerable risks, costs, and logistical hardship on those in remote communities – and is also a substantial contributor to healthcare’s carbon footprint (Hanneman et al., 2024b). For patients in remote regions, long-distance travel by air or road is often required, adding significant GHG emissions (Purohit et al., 2021). For example, a round trip between Weeneebayko General Hospital (WGH) in Moose Factory, Ontario, and Kingston Health Sciences Centre (KHSC) generates 879 kg of CO₂ emissions per patient for air transport alone (United States Environmental Protection Agency, 2024). Given that patients often require multiple imaging appointments, the cumulative environmental cost of transport is substantial (Figure 1).

**Figure 2:**
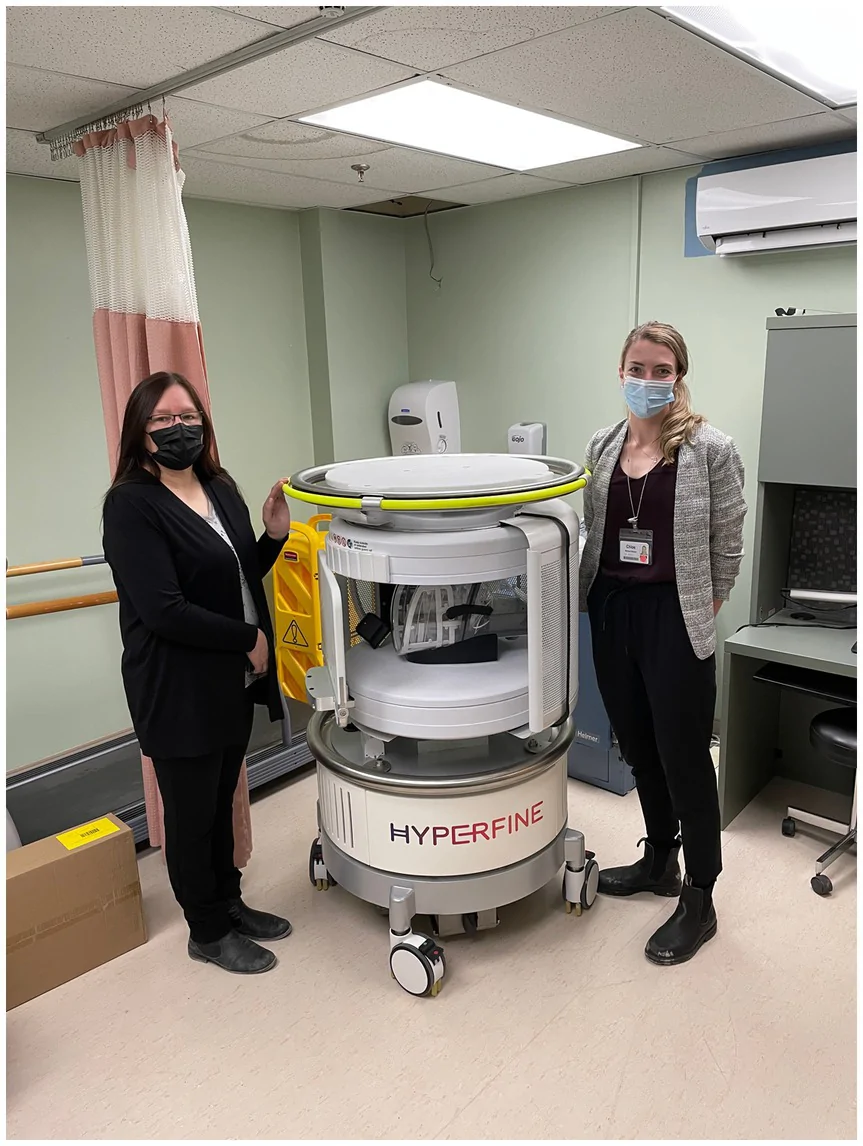
Portable MRI is an ultra-low-field bedside neuroimaging system that can be deployed in settings without conventional MRI infrastructure. The system can be housed in an existing clinical space, connected to standard power, and operated by trained local staff, with images transferred electronically for remote neuroradiology interpretation. Current pMRI systems are most useful for selected brain imaging indications, including screening or triage for larger intracranial abnormalities such as infarction, hemorrhage, hydrocephalus, mass effect, and midline shift. However, pMRI does not replace high-field MRI for all indications because of a lower signal-to-noise ratio, reduced spatial resolution, limited sequence availability, and the lack of routine contrast-enhanced, angiographic, perfusion, functional, or metabolic imaging. Portable MRI machine in Moose Factory with Dr. Elaine Innes (Chief of Staff, Weeneebayko Area Health Authority) and Chloe DesRoche (Queen’s University School of Medicine).

Recent advances in portable MRI (pMRI) technology offer a promising path forward. Multiple portable, ultra-low-field pMRI systems have emerged over the past decade – spanning commercially available, regulator-cleared devices and open-source designs – reflecting low-cost and low-infrastructure imaging, which can be moved into existing clinical spaces without conventional MRI infrastructure, connected to standard power, and operated by trained local staff. Images are transferred electronically for remote neuroradiologist interpretation. Early clinical studies highlight its usefulness across care settings, including the emergency department for rapid stroke triage and intensive care units where bedside imaging reduces the risks of moving critically ill patients (Islam et al., 2023; Bhat et al., 2021). Unlike conventional fixed MRI systems, pMRI does not require helium cooling, produces less electronic and structural waste, and avoids reliance on contrast agents that can contaminate water sources (Chaban et al., 2023). Its lower energy demands and reduced material requirements suggest that pMRI may substantially lessen the environmental burden of diagnostic imaging, while also expanding access to communities where traditional MRI is difficult to establish (Figure 2). However, pMRI cannot currently replace conventional MRI in all clinical scenarios. Its lower magnetic field strength limits spatial resolution and specialized applications, such as contrast-enhanced studies, vascular and perfusion assessment, and comprehensive body evaluation. Therefore, its greatest potential may lie in settings where it improves access and reduces unnecessary transfers.

Limited research has quantified the environmental impact of pMRI, particularly in remote settings where benefits could be most pronounced. While modelling studies suggest fixed MRI contributes up to 58.3 tons of CO₂ annually per scanner, equivalent to 14.6 kg of CO₂ per scan (Truhn et al., 2024), comprehensive analyses of the pMRI’s comparative performance remain scarce. Addressing this knowledge gap is essential to determine whether widespread adoption of pMRI could simultaneously improve diagnostic equity and advance planetary health goals.

This study incorporates clinical context from prior research to evaluate the environmental impact of pMRI implementation in a remote Canadian community. Specifically, we ask: (1) how does pMRI compare to fixed MRI in terms of carbon emissions associated with energy consumption; (2) how do the two compare in terms of air-transport-related carbon emissions; and (3) to what extent can pMRI reduce carbon emissions associated with waste generation? By integrating quantitative data on energy use, transportation emissions, and material waste, this study generates evidence to guide sustainable imaging practices and inform policy decisions supporting equitable healthcare delivery.

## Methods

### Study design

The environmental model is grounded in clinical results from the Desroche et al. (2024) feasibility study at WGH, which enrolled 25 consecutive patients referred for brain imaging with the 64 mT pMRI scanner. Indications were acute neurological presentations for which pMRI has established utility—suspected infarction, hemorrhage, hydrocephalus, mass effect, and midline shift—and all completed examinations were of diagnostic quality. Of the 25 patients, 14 (56%) were judged not to require inter-facility transfer to a fixed-MRI centre and thereby avoided a round-trip air journey generating approximately 879 kg of CO₂ per patient; the remaining 11 (44%) still required transfer for clinical presentations or imaging questions exceeding pMRI capabilities, such as high-resolution, contrast-enhanced, vascular, or functional MRI. When pMRI detected an abnormality warranting further characterization, transfer remained the clinically appropriate response—a positive patient outcome that is nonetheless environmentally negative—so the modelled savings apply only to the subset in whom pMRI was clinically sufficient, not to those whose findings appropriately prompted referral.

We modelled a standardized annual volume of 100 pMRI examinations, corresponding to the projected steady-state patient volume at WGH rather than early-pilot throughput. The feasibility examinations were performed over an approximately 10-month window (approximately 30 examinations/year), and the cost analysis projected approximately 50 examinations/year—the expected patient capacity in the first year of routine operation (Desroche et al., 2024). A five-year impact model extended this trajectory: under a gradual pMRI adoption scenario, eligible volume was assumed to rise from 50 examinations in year 1 to 75 in year 2 and 100 in years 3 through 5, matching the 100 eligible patients per year assumed throughout the fixed-MRI comparator scenario (Desroche et al., 2024). We therefore adopt 100 examinations as our annual scenario, representing the mature steady-state volume once pMRI transitions from a time-limited study to routine operation. Energy, transportation, and contrast-related emissions scale linearly with this volume and can be re-expressed for any assumed annual count; helium and end-of-life shielding emissions are per-scanner lifecycle values, independent of annual scan volume. Under the 56% avoided-transfer estimate above, 56 of every 100 modelled examinations avoid an inter-facility round trip.

This study employed a mixed-methods approach to evaluate energy usage, transportation-related GHG emissions, and waste generation associated with the 64 mT pMRI scanner. We evaluated the 64 mT system WGB because it is, to our knowledge, currently the only portable MRI that is both Health Canada-licensed and commercially available for clinical brain imaging. Other portable low-field platforms are not yet suited to this use: the OSI (Hanneman et al., 2024a) ONE is an open-source research system rather than a commercial, regulator-cleared device, and PhysioMRI is a commercial system oriented toward extremity imaging, with brain applications still investigational and without Health Canada approval. This study had two primary objectives: (1) to quantify and compare energy usage and its associated CO₂ emissions between pMRI and conventional fixed MRI systems; assess air-transport-related CO₂ emissions from the implementation of pMRI at WGH; and (2) to assess waste generation associated with both modalities, expressed in terms of avoided CO₂ emissions. This study focuses on the population served by the Weeneebayko Area Health Authority (WAHA), which provides care to approximately 12,000 predominantly Indigenous people across the remote James Bay and Hudson Bay coastal communities and includes WGH in Moose Factory. WGH has on-site CT and ultrasound but no conventional MRI; patients currently requiring MRI scans are therefore transferred to fixed-MRI centres such as Kingston (KHSC) or Timmins (Desroche et al., 2024). This study did not involve human subjects or patient-level data, so research ethics board approval was not required in accordance with TCPS2, Article 2.1.

### Objective 1: energy usage and transportation emissions

A literature search of Embase and PubMed identified English-language original research and review articles published from 2014 onward, using the terms MRI, portable MRI, ultra-low-field MRI, energy, power, cost, and feasibility study. Articles were screened for relevance and methodological quality, and the extracted data were used to compare energy use, carbon emissions, and cost across modalities. Energy use for pMRI was converted to CO₂ equivalents using the Government of Canada’s Greenhouse Gas Equivalencies Calculator (Natural Resources Canada, 2025) and compared with published values for conventional 1.5 Tesla (T) and 3 T scanners.

For transportation, CO₂ emissions were estimated for the shortest air route between Moose Factory and Kingston. Because the WGH catchment communities are accessible only by air, ground transport was not modelled. Patients requiring transfer were distributed across the three air modes serving WGH—commercial (64%), charter (23%), and air ambulance (13%), based on WAHA/NIHB medical-transport records for this route, with the assigned mode reflecting clinical urgency and local availability. Aircraft-specific, per-passenger emission factors for each mode (Table 1), obtained from the U.S. Environmental Protection Agency, were applied to this distribution to estimate the emissions avoided through local pMRI access.

**Table 1:** CO_2_ coefficient per vehicle type.

	Vehicle/km	Passenger/km
Vehicle type	CO_2_ factor (kg CO_2_/ km)	CO_2_ factor (kg CO_2_/km)
De Havilland DHC-8	1.73	0.12
Cessna 208 Caravan	1.73	0.19
Pilatus PC-12	1.18	0.13
Leonardo AW139	5.25	0.35

### Objective 2: waste generation and associated CO₂ emissions

This analysis focused on three categories of MRI-related waste: (1) end-of-life disposal, (2) helium consumption, and (3) contrast agent waste. We searched the existing literature on the environmental waste of traditional MRIs. Due to the novelty of pMRI technology, quantifiable data on its waste were limited. To better understand waste generation in pMRI, we examined individual components of pMRI technologies such as Hyperfine’s Swoop and compared them to current fixed MRI systems. Additionally, to calculate associated CO₂ emissions from waste, calculations were done based on current available data in the literature. To analyze end-of-life disposal, copper RF shielding requirements for a typical 1.5 T MRI suite were combined with cradle-to-gate GHG intensities for refined copper to estimate embodied emissions (Gan et al., 2023). To determine helium consumption, lifetime helium use for superconducting MRI was estimated with liquefaction. Emissions were calculated using Ontario and Canadian average grid intensities (Mahesh and Barker, 2016). Life-cycle assessment data were used to estimate CO₂e (CO₂ emitted) per administration from single-use contrast agent infusion devices. Additionally, the volume of gadolinium discharged annually into U.S. wastewater, where gadolinium-based contrast agents persist in aquatic systems, was calculated based on currently available online data (Brünjes and Hofmann, 2020; Ebrahimi and Barbieri, 2019).

## Results

### CO₂ emissions from energy usage of pMRI versus fixed MRI

The 64 mT scanner has a maximum power demand of 1,650 W for the entire system, including the console, radiofrequency power amplifier, and three gradient amplifiers (Cooley et al., 2021). By contrast, conventional clinical MRI units require substantially greater energy input. Heye and colleagues (2020) reported daily energy consumption of 363 kWh for 1.5 T systems and 530 kWh for 3 T systems, corresponding to a mean of 19.9 kWh per examination. Furthermore, Truhn and colleagues (2024) estimated that a 3 T MRI operating over one year produces approximately 58.3 tons of CO₂, or 14.6 kg of CO₂ per scan. pMRI devices, however, generate markedly fewer GHG emissions. The 64 mT scanner consumes just 1.65 kWh per hour of operation—approximately 11.8-fold less than a 1.5 T MRI (15.9 kWh per 49-min head exam) and 17.4-fold less than a 3 T MRI (23.9 kWh per 50-min head exam). Based on 100 h of annual operation, reflecting our model of 100 scans (1 h per scan), total yearly energy use amounts to only 165 kWh. This translates to 0.065 metric tons of CO₂ emissions—equivalent to burning 27.6 L of gasoline—compared with 266.5 L for a 1.5 T MRI and 401.3 L for a 3 T system (United States Environmental Protection Agency, 2024).

### CO₂ emissions from patient transportation to remote community

Implementation of pMRI is estimated to substantially reduce carbon emissions in the WAHA communities compared to scenarios without pMRI utilization by replacing, for selected patients, transfer to a tertiary or regional fixed-MRI centre. Eliminating a round-trip air journey between WGH and KGH for 56% of patients would reduce emissions by approximately 7.23 tons (t) from commercial flights, 4.04 t from charter flights, and 3.04 t from air ambulance flights. The total estimated reduction is 14.31 t of CO₂ per year (Table 2), which is equivalent to the emissions produced by approximately 1.9 single-family homes annually, as per EPA calculations (United States Environmental Protection Agency, 2024). Commercial and charter flights together accounted for 78.6% of modelled travel-related CO₂ emissions annually, compared to 21.2% from emergent flights. For air ambulance transport, a medium-sized twin-engine helicopter (Leonardo AW139) had a 2.66-fold higher per-passenger emission than a single-engine turboprop Pilatus PC-12 aircraft. A charter flight (Cessna 208 Caravan) had a 1.66-fold higher CO₂ emission per passenger compared to commercial flights (De Havilland DHC-8). Overall, the medium-sized twin-engine helicopter (Leonardo AW139) emitted the most CO₂ per passenger (Table 3).

**Table 2:** Average amount of CO_2_ emitted per flight type/year for 56 patients.

Transportation type	AVG CO_2_ factor (kg CO_2_/km/passenger)	CO_2_e/passenger (round-trip)	AVG # of patients/year	t CO_2_e	Total t CO_2_e/year
Commercial flight	0.12	201.84	35.82	7.23	14.31
Charter flight	0.19	319.58	12.64	4.04	
Emergent flight	0.24	403.68	7.52	3.04	

**Table 3:** Total amount of CO_2_ emitted (kg) per passenger/vehicle type between Moose Factory and Kingston, ON.

Air travel (WGH-KGH)	One-way (km)	Round-trip (km)
Commercial flight		
De havilland DHC-8	100.92	193.71
Charter flight		
Cessna 208 caravan	161.19	322.38
Orgne air ambulance		
Pilatus PC-12	110.36	220.72
Leonardo AW139	294.08	588.17

### End-of-life disposal for pMRI versus fixed MRI

Fixed MRIs employ superconducting magnets (e.g., Nb–Ti or Nb₃Sn wire in copper) that require liquid helium cooling, creating significant disposal challenges due to heavy metal content (Chaban et al., 2023; Pantalony, 2011). pMRI, by contrast, uses permanent neodymium–iron–boron magnets that are lighter, helium-free, and more easily recyclable (Huang et al., 2019). Both modalities incorporate RF coils and electronic circuits containing copper, printed circuit boards, and heavy metals such as lead and tin, all requiring regulated disposal (Kimberly et al., 2023). However, fixed MRIs generate substantially greater electronic waste due to their larger size and the extensive shielding required, often composed of copper, lead, or steel (American Institute of Physics, 1987). For example, a typical 1.5 T MRI suite requires ~1,500 kg of copper shielding, corresponding to ~5.0–7.2 t CO₂e of embodied emissions, equivalent to the annual emissions of 0.67–0.97 single-family homes. By operating at lower field strengths, portable systems generally require minimal shielding and incorporate built-in RF noise cancellation, thereby reducing decommissioning waste and avoiding these carbon costs (Gan et al., 2023).

### Helium consumption in fixed MRI systems

Superconducting MRI systems consume large volumes of liquid helium, with lifespans requiring up to 10,000 L and ~2,000 L contained in the unit at any one time (Mahesh and Barker, 2016). Loss occurs through boil-off, quenching, and limited recycling capacity, contributing to depletion of a finite global resource (Chaban et al., 2023). In 2017, MRI operations accounted for ~30% of U.S. helium use. Helium liquefaction is energy-intensive, generating ~0.4–2.6 t CO₂e on the Ontario grid over a scanner’s lifespan—approximately the annual emissions of 1.2 home equivalents (United States Environmental Protection Agency, 2024). Portable MRI units use permanent magnets and require no helium, fully eliminating this source of waste and associated carbon emissions.

### Contrast agent waste in fixed MRI systems

Conventional MRI frequently employs gadolinium-based contrast agents (GBCAs), which generate residual waste from syringes, tubing, and expired stock (Robinson et al., 2013; Serai et al., 2021). Nearly all administered gadolinium is excreted and enters wastewater, with ~21 t discharged annually in the United States (Brünjes and Hofmann, 2020). GBCAs are chemically stable, resisting degradation, accumulate in aquatic systems, and can enter food chains (Ebrahimi and Barbieri, 2019). Health concerns from GBCAs include nephrogenic systemic fibrosis and gadolinium deposition, even in patients with normal renal function (Bendszus et al., 2024). From a carbon perspective, each GBCA administration produces ~0.28–0.56 kg CO₂e from single-use devices and packaging.

In practice, most pMRI scans are performed without contrast because the low-field strength limits the effectiveness of gadolinium, as these systems are optimized for basic bedside imaging. Substituting pMRI for 100 contrast-enhanced exams would therefore prevent ~28–56 kg CO₂e annually, equivalent to the emissions from burning 43.9–87.4 L of gasoline (United States Environmental Protection Agency, 2024).

Replacing conventional fixed MRI systems with pMRI could prevent ~5.4–10 t CO₂e per scanner when accounting for end-of-life shielding and helium consumption, in addition to ~28–56 kg CO₂e annually saved by avoiding contrast-related waste for every 100 exams (United States Environmental Protection Agency, 2024). Collectively, these findings underscore the environmental advantages of pMRI, not only by reducing operational energy demands but also by minimizing material, cryogen, and medical waste across the imaging lifecycle.

### Total combined carbon emissions from energy, transportation, and waste generation

Based on calculations, carbon emissions from energy usage were negligible, with transportation contributing the most carbon emissions behind waste generation and its associated carbon emissions (Table 4).

**Table 4:** Carbon emission comparison between fixed MRI and pMRI for energy consumption, air travel, and waste generation.

	Fixed MRI		pMRI	
	CO_2_e (Kg)/Patient	Total CO_2_e (kg)	CO_2_e (Kg)/Patient	Total CO_2_e (kg)
Energy consumption (*N* = 100)	14.6	1,460	N/A	N/A
Energy equivalent gasoline consumed avoided (L)		**266.5–401.3 L**		
Air transportation (round trip between Moose Factory and Kingston, ON)				
Commercial (*N* = 35.8)	N/A	N/A	201.8	7225.9
Charter (*N* = 12.6)	N/A	N/A	319.6	4026.7
Emergent (*N* = 7.5)	N/A	N/A	403.7	3027.6
Total CO_2_ saved from air transport avoidance for 56/100 patients				14280.2
Transportation home equivalent energy use/year				1.9
Waste generation				
End-of-life disposal (lifetime)	N/A	5,000–7,200	N/A	N/A
Helium consumption (lifetime)	N/A	400–2,600	N/A	N/A
Contrast agent waste (*N* = 100)	0.28–0.56	28–56	N/A	N/A
Waste generation home equivalent energy use/year		5,400–9,800		0.73–1.3
Total home equivalents				2.6–3.2

## Discussion and conclusion

This study is a comprehensive evaluation of multiple environmental dimensions—energy, transport, and waste—modelled after a real-world remote northern Canadian community. Building on prior feasibility work showing that diagnostic-quality neuroimaging can be obtained locally with pMRI (Desroche et al., 2024), this study modelled the environmental implications of that care pathway and found that local pMRI substantially reduces the carbon footprint of neuroimaging relative to a conventional fixed-MRI pathway that requires inter-facility transfer. These environmental benefits are best understood as a co-benefit of a technology whose primary value is improving health equity and local diagnostic access for remote communities; the environmental advantage follows from, and is contingent on, the clinical role.

### Energy efficiency in context

Previous work has shown that fixed MRI is one of the most energy-intensive modalities in radiology, with operational demands that exceed many other diagnostic technologies (Heye et al., 2020; Truhn et al., 2024). In contrast, pMRI operates on standard electrical outlets and does not require dedicated cooling or specialized infrastructure (Cooley et al., 2021). The comparative analyses in this study showed that pMRIs consume considerably less energy. Clinically, pMRI has already proven useful in emergency and critical care settings, where rapid, bedside neuroimaging is required (Islam et al., 2023; Bhat et al., 2021). The findings here extend this evidence by showing that pMRI adoption also aligns with national and institutional carbon reduction goals, with a fraction of the energy required by conventional systems.

### Transportation as a dominant factor

Across the dimensions assessed, avoiding long-distance patient travel was the largest contributor to environmental benefit—consistent with evaluations of telemedicine and virtual care, in which preventing unnecessary transport yields the greatest carbon savings (Purohit et al., 2021). Local pMRI reduces not only emissions but also the risks, costs, and logistical burden of transferring patients from remote communities to tertiary centres. This benefit, however, is directional and case-mix dependent: it arises specifically when pMRI triages patients away from transfer, and it does not assume that pMRI replaces all fixed MRI. When pMRI instead detects an abnormality that would otherwise have remained undiscovered, it may appropriately prompt a new transfer—a desirable clinical outcome that nonetheless adds rather than avoids travel. pMRI is therefore best positioned for local triage and selected diagnostic pathways, with transfer reserved for patients who require higher-resolution, contrast-enhanced, vascular, perfusion, or otherwise advanced imaging (Desroche et al., 2024).

### Scalability of carbon savings

The savings modelled here reflect a single small catchment and are correspondingly modest in absolute terms. Still, the driver of those savings—averted long-distance medical transport—applies across many comparable settings. The community modelled is served by a health authority of roughly 12,000 people, for which prior work projected on the order of 50 neuroimaging-eligible pMRI examinations per year (Desroche et al., 2024). Northern Ontario’s remote, fly-in First Nations are considerably larger in aggregate: about 22,400 on-reserve residents live in the 26 Nishnawbe Aski Nation communities without year-round road access, from which roughly 2,100 emergency air-transports are undertaken annually (about 9 per 100 residents), with neurological presentations accounting for roughly one in ten (VanderBurgh et al., 2020). Nationally, the transport burden is larger still—Canada’s territories alone generate tens of thousands of medical-travel trips each year, with medevac rates of 32–53 per 1,000 residents in the Northwest Territories and Nunavut and medical travel consuming roughly 5–20% of territorial health budgets (Young et al., 2019). Only a subset of this transport is amenable to substitution by local pMRI—chiefly non-urgent neuroimaging for which ultra-low-field imaging is diagnostically adequate—so these figures represent the scale of transport-dependence rather than a pool of fully avoidable trips. Even so, they indicate that modest per-site savings, replicated across the dozens of remote communities that rely on long-distance transport provincially and nationally, could aggregate into substantial reductions in both emissions and cost. Robust province- or country-level estimates will require site-specific catchment, case-mix, and aviation data rather than extrapolation from a single program.

### Waste and materials

Another advantage of pMRI is its streamlined material profile. Unlike conventional superconducting systems, pMRI requires no liquid helium and minimal radiofrequency shielding, reducing dependence on scarce resources and lowering end-of-life waste. Furthermore, the avoidance of gadolinium-based contrast agents eliminates a source of medical waste and prevents the release of persistent contaminants into water systems (Brünjes and Hofmann, 2020; Ebrahimi and Barbieri, 2019). Although these impacts are smaller than transport-related emissions, their importance lies in removing entire categories of waste that pose both environmental and supply-chain challenges.

### Limitations

Limitations include: (i) reliance on literature-derived energy and travel emission factors rather than continuous on-device metering or ticket-level flight data; (ii) uncertainty in annual scan volumes and case mix, which can shift per-scan energy use and travel-related emission savings; and (iii) limited generalizability beyond the study region, as aviation modes, distances, and community referral patterns vary; and (iv) the local energy estimate does not capture off-site (cloud-based) reconstruction compute, although this per-scan increment is small relative to the modality difference and to the transport-related emissions that dominate the analysis in this study.

The energy and material values reported here are specific to the ultra-low-field system evaluated; other portable platforms differ in magnet configuration, power electronics, construction, and would carry different profiles. Other comparable systems for brain imaging are currently investigational or open-source; therefore, a head-to-head environmental comparison is not yet feasible, and these estimates should be read as representative of ultra-low-field pMRI rather than universal figures.

A limitation of pMRI is its diagnostic scope. In the underlying feasibility cohort, all completed examinations were considered diagnostic quality, although 5 of 25 carried non-optimal quality comments (motion or zipper artifact, suboptimal head positioning), and 44% of patients would still require transfer for fixed MRI because of clinical presentation or resolution limits (Desroche et al., 2024). This aligns with the known constraints of ultra-low-field imaging—lower signal-to-noise ratio, reduced spatial resolution, longer acquisition times, limited sequence availability, and the absence of routine contrast-enhanced, angiographic, perfusion, functional, or metabolic imaging (Islam et al., 2023; Desroche et al., 2024; Altaf et al., 2024). Environmental benefits should therefore be interpreted alongside clinical safeguards so that avoided transfers do not compromise diagnostic adequacy. Relatedly, contrast-related waste savings warrant caution: the feasibility cohort did not establish that all transferred patients would have received gadolinium, and many common indications (acute stroke, head injury, dizziness, numbness or tingling) use non-contrast protocols. These savings therefore apply mainly where pMRI substitutes for an examination that would otherwise have required contrast-enhanced MRI.

### Future directions

This study highlights the need for further research into several areas. Future work should include multi-site LCAs comparing 64 mT, 1.5 T, and 3 T MRI across rural/remote and urban programs, incorporating cradle-to-grave accounting of off-site (cloud) reconstruction compute. Additional studies should evaluate how integrating teleconsultation models and pMRI workflows can maximize avoided travel while maintaining diagnostic quality. Standardized aviation accounting across commercial, charter, and rotary wing transport models would improve comparability between studies. Finally, investigations into material circularity, including electronic component reuse and rare-earth magnet recycling, are needed to better quantify end-of-life environmental impacts. Prospective LCAs incorporating site-specific electricity mixes, real utilization data, and cradle-to-grave materials accounting should be paired with clinical outcome measures, including diagnostic adequacy and downstream re-imaging rates, to ensure that environmental benefits are achieved without compromising care quality.

## Conclusion

**Figure 3:**
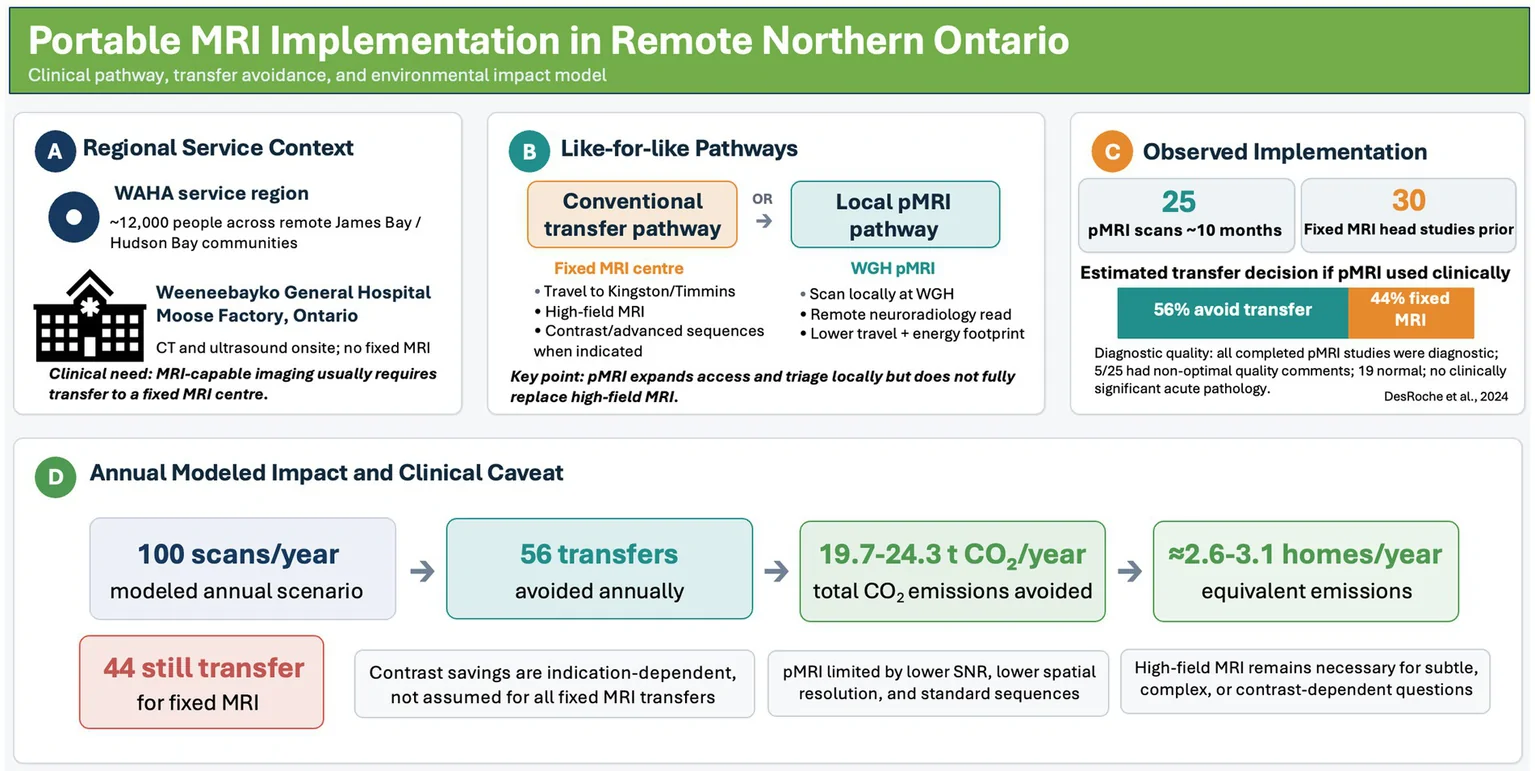
Clinical pathway and environmental model for portable MRI implementation in a remote Canadian setting. **(A)** Weeneebayko Area Health Authority (WAHA) serves approximately 12,000 people across remote James Bay and Hudson Bay communities. Weeneebayko General Hospital (WGH) has CT and ultrasound onsite but no fixed MRI. **(B)** The conventional pathway requires patient transfer to a fixed MRI centre such as Kingston or Timmins. In contrast, the local pMRI pathway allows selected neuroimaging to be performed at WGH with remote interpretation. **(C)** In the feasibility cohort, 25 pMRI scans were performed over approximately 10 months; during the comparable prior period, 30 fixed MRI head studies were performed for WGH patients. Based on clinical presentation and pMRI findings, 56% of patients could avoid transfer, while 44% would still require fixed MRI. **(D)** Applying this avoided-transfer proportion to a modelled annual scenario of 100 scans, pMRI could avoid 56 transfers per year and reduce travel-related emissions by approximately 19.7–24.3 t CO_2_/year, equivalent to emissions from approximately 2.6–3.1 single-family homes. The 100 scans/year value represents a modelled scenario, not the observed feasibility cohort. Data are based on the provided feasibility and scoping-review articles. Abbreviations: WAHA, Weeneebayko Area Health Authority; WGH, Weeneebayko General Hospital: pMRI, portable magnetic resonance imaging: SNR, signal-to-noise ratio. The 100 scans/year value is a modelled scenario, distinct from the observed feasibility cohort in panel 3.

In a remote Canadian setting, portable MRI has been shown to provide diagnostic-quality neuroimaging for a predominantly Indigenous population and to allow 56% of patients to avoid inter-facility transfer to a tertiary centre (Desroche et al., 2024). Building on those clinical findings, the present study modelled the environmental implications of that care pathway and found a substantially lower carbon footprint than a fixed-MRI pathway—through lower operational energy without dedicated cooling, avoided high-carbon inter-facility travel for patients who do not require transfer, and elimination of helium and contrast-related waste streams—totalling roughly 2.6–3.1 single-family-home-years of carbon avoided annually at the scan volumes modelled (Figure 3). These environmental gains are a co-benefit of pMRI’s primary clinical rationale and are bounded by the modest volumes of a single program; their broader significance lies in the potential to scale across the many remote communities that depend on long-distance medical transport. Wider adoption should proceed alongside rigorous prospective LCAs and clinical-quality metrics; on current evidence, pMRI represents a pragmatic, high-impact means of advancing equitable neuroimaging access in remote and resource-limited settings while simultaneously reducing the environmental footprint of care.

## Data Availability

The original contributions presented in the study are included in the article/supplementary material, further inquiries can be directed to the corresponding author.
